# The US Department of Veterans Affairs Science and Health Initiative to Combat Infectious and Emerging Life-Threatening Diseases (VA SHIELD): A Biorepository Addressing National Health Threats

**DOI:** 10.1093/ofid/ofac641

**Published:** 2022-12-14

**Authors:** John B Harley, Saiju Pyarajan, Elizabeth S Partan, Lauren Epstein, Jason A Wertheim, Abhinav Diwan, Christopher W Woods, Victoria Davey, Sharlene Blair, Dennis H Clark, Kenneth M Kaufman, Shagufta Khan, Iouri Chepelev, Alexander Devine, Perry Cameron, Monica F McCann, Mary Cloud B Ammons, Devin D Bolz, Jane K Battles, Jeffrey L Curtis, Mark Holodniy, Vincent C Marconi, Charles D Searles, David O Beenhouwer, Sheldon T Brown, Jonathan P Moorman, Zhi Q Yao, Maria C Rodriguez-Barradas, Shyam Mohapatra, Osmara Y Molina De Rodriguez, Emerson B Padiernos, Eric R McIndoo, Emily Price, Hailey M Burgoyne, Ian Robey, Dawn C Schwenke, Carey L Shive, Ronald M Przygodzki, Rachel B Ramoni, Holly K Krull, Robert A Bonomo

**Affiliations:** Research Services, US Department of Veterans Affairs Medical Center, Cincinnati, Ohio, USA; Center for Data and Computational Sciences, Veterans Affairs Boston Healthcare System, Boston, Massachusetts, USA; Center for Data and Computational Sciences, Veterans Affairs Boston Healthcare System, Boston, Massachusetts, USA; Infectious Diseases, US Department of Veterans Affairs Medical Center, Atlanta, Georgia, USA; Research & Development, Southern Arizona Veterans Affairs Healthcare System, US Department of Veterans Affairs, Tucson, Arizona, USA; Cardiology, Veterans Affairs Saint Louis Healthcare System, US Department of Veterans Affairs, Saint Louis, Missouri, USA; Medicine, US Department of Veterans Affairs Medical Center, Durham, North Carolina, USA; Office of Research and Development, US Department of Veterans Affairs, Washington, District of Columbia, USA; Research Services, US Department of Veterans Affairs Medical Center, Cincinnati, Ohio, USA; Research Services, US Department of Veterans Affairs Medical Center, Cincinnati, Ohio, USA; Research Services, US Department of Veterans Affairs Medical Center, Cincinnati, Ohio, USA; Research Services, US Department of Veterans Affairs Medical Center, Cincinnati, Ohio, USA; Research Services, US Department of Veterans Affairs Medical Center, Cincinnati, Ohio, USA; Prometheus Federal Services, Titan Alpha, Washington, District of Columbia, USA; Customer Value Partners, Titan Alpha, Washington, District of Columbia, USA; Office of Research and Development, Chesapeake Medical Communications, Contractor for the US Department of Veterans Affairs, Washington, District of Columbia, USA; Research, US Department of Veterans Affairs Medical Center, Boise, Idaho, USA; Idaho Veterans Research and Education Foundation, Boise, Idaho, USA; Research, US Department of Veterans Affairs Medical Center, Boise, Idaho, USA; Office of Research and Development, US Department of Veterans Affairs, Washington, District of Columbia, USA; Medicine Service, Veteran Affairs Ann Arbor Healthcare System, US Department of Veterans Affairs, Ann Arbor, Michigan, USA; Public Health Surveillance, Veterans Affairs Palo Alto Healthcare System, US Department of Veterans Affairs, Palo Alto, California, USA; Infectious Diseases, US Department of Veterans Affairs Medical Center, Atlanta, Georgia, USA; Division of Infectious Diseases, Emory School of Medicine and Rollins School of Public Health, Atlanta, Georgia, USA; Infectious Diseases, US Department of Veterans Affairs Medical Center, Atlanta, Georgia, USA; Medicine, Veterans Affairs Greater Los Angeles Healthcare System, US Department of Veterans Affairs, Los Angeles, California, USA; Infectious Diseases, James J. Peters Veterans Affairs Medical Center, US Department of Veterans Affairs, Bronx, New York, USA; Infectious Diseases, James H. Quillen Veterans Affairs Medical Center, US Department of Veterans Affairs, Mountain Home, Tennessee, USA; Center of Excellence in Inflammation, Infectious Diseases, and Immunity, East Tennessee State University, Johnson City, Tennessee, USA; Infectious Diseases, James H. Quillen Veterans Affairs Medical Center, US Department of Veterans Affairs, Mountain Home, Tennessee, USA; Center of Excellence in Inflammation, Infectious Diseases, and Immunity, East Tennessee State University, Johnson City, Tennessee, USA; Infectious Diseases Section, Michael E. DeBakey Veterans Affairs Medical Center, US Department of Veterans Affairs, Houston, Texas, USA; Department of Medicine, Baylor College of Medicine, Houston, Texas, USA; Medicine, James A. Haley Veterans Hospital, US Department of Veterans Affairs, Tampa, Florida, USA; Research & Development, Southern Arizona Veterans Affairs Healthcare System, US Department of Veterans Affairs, Tucson, Arizona, USA; Research, US Department of Veterans Affairs Medical Center, Boise, Idaho, USA; Research, US Department of Veterans Affairs Medical Center, Boise, Idaho, USA; Idaho Veterans Research and Education Foundation, Boise, Idaho, USA; Research, US Department of Veterans Affairs Medical Center, Boise, Idaho, USA; Idaho Veterans Research and Education Foundation, Boise, Idaho, USA; Research, US Department of Veterans Affairs Medical Center, Boise, Idaho, USA; Idaho Veterans Research and Education Foundation, Boise, Idaho, USA; Research & Development, Southern Arizona Veterans Affairs Healthcare System, US Department of Veterans Affairs, Tucson, Arizona, USA; Research & Development, Southern Arizona Veterans Affairs Healthcare System, US Department of Veterans Affairs, Tucson, Arizona, USA; Medicine, Veterans Affairs Northeast Ohio Healthcare System, US Department of Veterans Affairs, Cleveland, Ohio, USA; Office of Research and Development, US Department of Veterans Affairs, Washington, District of Columbia, USA; Office of Research and Development, US Department of Veterans Affairs, Washington, District of Columbia, USA; Office of Research and Development, US Department of Veterans Affairs, Washington, District of Columbia, USA; Medicine, Veterans Affairs Northeast Ohio Healthcare System, US Department of Veterans Affairs, Cleveland, Ohio, USA; Departments of Medicine, Pharmacology, & Molecular Biology and Microbiology, Case Western Reserve University, Cleveland, Ohio, USA

**Keywords:** biological specimen banks, communicable diseases, COVID-19 sequence analysis, data warehousing, government agencies, health policy, public health surveillance, Veterans health

## Abstract

**Background:**

The coronavirus disease 2019 (COVID-19) pandemic, caused by the severe acute respiratory syndrome coronavirus 2 (SARS-CoV-2), has demonstrated the need to share data and biospecimens broadly to optimize clinical outcomes for US military Veterans.

**Methods:**

In response, the Veterans Health Administration established VA SHIELD (Science and Health Initiative to Combat Infectious and Emerging Life-threatening Diseases), a comprehensive biorepository of specimens and clinical data from affected Veterans to advance research and public health surveillance and to improve diagnostic and therapeutic capabilities.

**Results:**

VA SHIELD now comprises 12 sites collecting de-identified biospecimens from US Veterans affected by SARS-CoV-2. In addition, 2 biorepository sites, a data processing center, and a coordinating center have been established under the direction of the Veterans Affairs Office of Research and Development. Phase 1 of VA SHIELD comprises 34 157 samples. Of these, 83.8% had positive tests for SARS-CoV-2, with the remainder serving as contemporaneous controls. The samples include nasopharyngeal swabs (57.9%), plasma (27.9%), and sera (12.5%). The associated clinical and demographic information available permits the evaluation of biological data in the context of patient demographics, clinical experience and management, vaccinations, and comorbidities.

**Conclusions:**

VA SHIELD is representative of US national diversity with a significant potential to impact national healthcare. VA SHIELD will support future projects designed to better understand SARS-CoV-2 and other emergent healthcare crises. To the extent possible, VA SHIELD will facilitate the discovery of diagnostics and therapeutics intended to diminish COVID-19 morbidity and mortality and to reduce the impact of new emerging threats to the health of US Veterans and populations worldwide.

## Teaser

The US Department of Veterans Affairs has implemented a biorepository providing biological specimens and data for studies of COVID-19, while simultaneously preparing for subsequent emerging life-threatening diseases. These resources are available for investigators to study COVID-19 and future emerging diseases.

Every aspect of the United States (US) healthcare enterprise has experienced substantial impacts from the coronavirus disease 2019 (COVID-19) pandemic. The US Department of Veterans Affairs (VA), through its Veterans Health Administration (VHA), has provided care for a large number of afflicted Veterans, which has taught us much [1], improving the morbidity and mortality of this serious illness.

At present, the VA addresses the healthcare needs of nearly 9 million US Veterans. As one of the largest integrated US healthcare systems, the VA provides outpatient care, hospitalization services, nursing home residence, and national cemeteries for America's heroes. A large number of US Veterans under VHA care were rapidly immunized by the highly effective VA severe acute respiratory syndrome coronavirus 2 (SARS-CoV-2) vaccination campaign in 2021 [2]. Nevertheless, the appearance of SARS-CoV-2 variants, combined with waning immunity postinfection or vaccination and postacute sequelae of SARS-CoV-2 infection (referred to as PASC), has created a need to address COVID-19 as an endemic disease.

VA leadership has responded to the COVID-19 pandemic challenge with initiatives designed to inform public policy and to improve management and therapeutics. One such initiative, VA SHIELD (the VA Science and Health Initiative to Combat Infectious and Emerging Life-threatening Diseases), is a comprehensive biorepository of specimens with accompanying clinical data, serving to advance research and improve diagnostic and therapeutic capabilities (Table 1). VA SHIELD is a national response to the current pandemic, while also poised to respond to future challenges to our healthcare system.

**Table 1.** Veterans Affairs Science and Health Initiative to Combat Infectious and Emerging Life-threatening Diseases (VA SHIELD) Purpose and Commitments

VA SHIELD Purpose	VA SHIELD Commitments
Develop a geographically representative biorepository that leverages the VA EHR to facilitate improved diagnostic, therapeutic, and prevention strategies Increase scientific impact and integrate VA research efforts Promote collaboration (internal and external to VA) Provide infrastructural and foundational support to facilitate research Serve as a premier resource for scientific and health research data that can be used by VA entities and investigators in future research studies	Maximize collection of high-quality specimens and data, ethically and efficiently Harness clinical and scientific strengths of the VA to be synergistic with preexisting efforts Provide an effective mechanism to review, approve, and distribute specimens and data to scientific partners Leverage the administrative and regulatory capabilities of VA to ensure accountability and compliance with applicable laws and regulations

Abbreviations: EHR, electronic health record; SHIELD, Science and Health Initiative to Combat Infectious and Emerging Life-threatening Diseases; VA, Veterans Affairs.

The VA SHIELD infrastructure also provides important additional advantages including opportunities to train the next generation of investigators in basic and public health–based research. The unique strengths of the VA healthcare system provide the capacity to collect biospecimens and associated data on an unprecedented scale. The work conducted with the biospecimens and data in VA SHIELD will be representative of national diversity and will have broad potential impact on national healthcare. Indeed, VA SHIELD improves our preparedness for responses to pandemics overall, thereby serving the Fourth Mission of the Veterans Health Administration: to serve society as a whole.

##  

### VA SHIELD: Structure and Oversight

**Figure 1.**
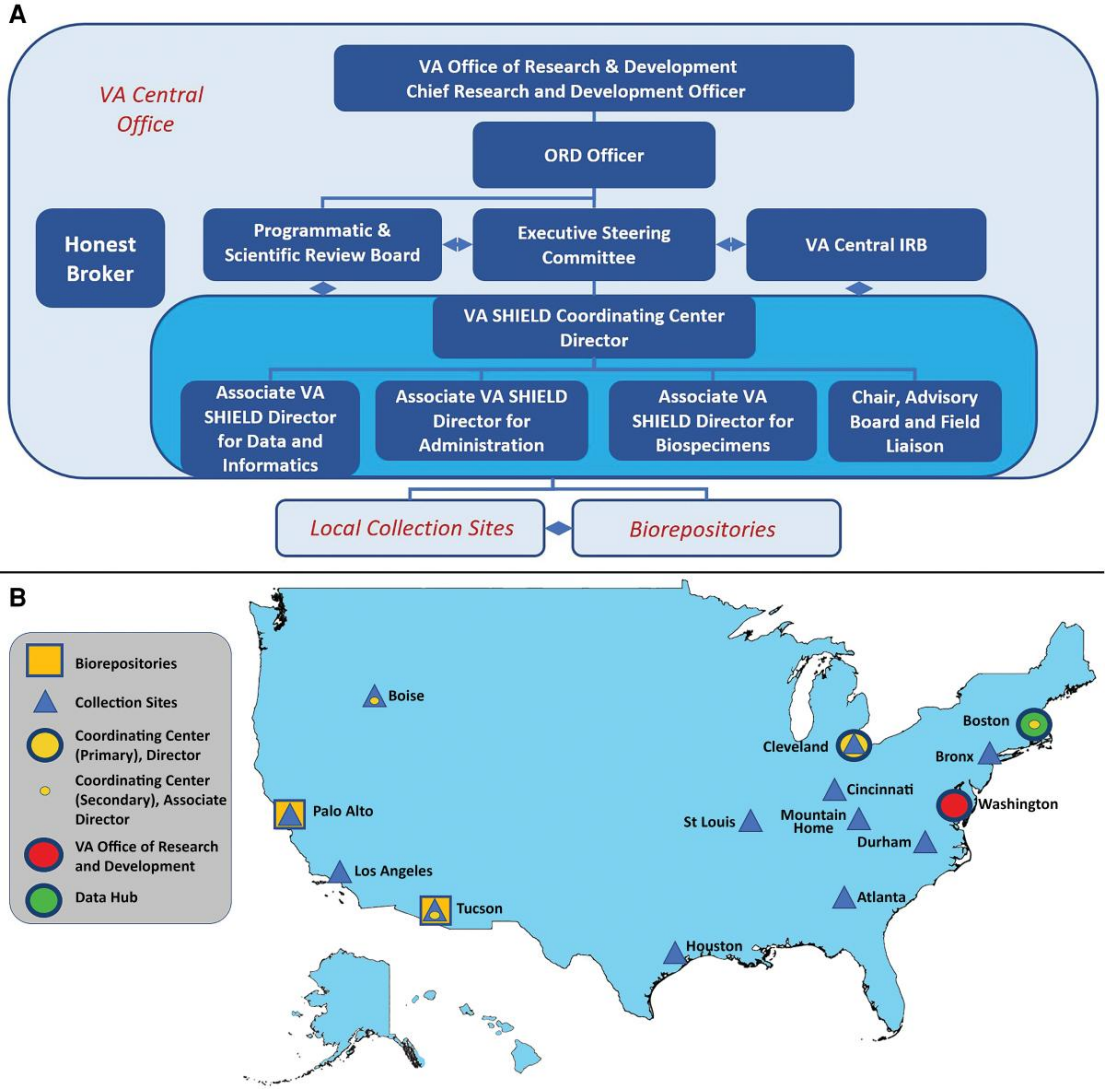
Governance structure and locations for the Veterans Affairs (VA) Science and Health Initiative to Combat Infectious and Emerging Life-threatening Diseases (SHIELD). VA SHIELD is nationally organized to create a comprehensive biospecimen repository with links to critical patient information and data. Under the guidance of the VA Office of Research and Development, the Coordinating Center provides oversight to collection sites by establishing standard operating procedures and providing the infrastructure needed to respond to emerging threats. Data from the VA electronic health record are associated with the biological samples by a newly designed laboratory information management system under the direction of VA SHIELD Data Processing Center. The long-term goal is to expand from these initial sites and to help best coordinate and respond to national health emergencies. The governance structure is presented in the upper panel (*A*) and the participating VA components in the lower panel (*B*). Abbreviations: IRB, institutional review board; ORD, Office of Research and Development; SHIELD, Science and Health Initiative to Combat Infectious and Emerging Life-threatening Diseases; VA, Veterans Affairs.

VA SHIELD operates within the VA Office of Research and Development (ORD) under management of the Chief Research and Development Officer (CRADO). The VA SHIELD ORD Officer, from the ORD leadership staff, is the liaison between VA SHIELD and the CRADO (Figure 1). In its current form, VA leadership is advised of matters related to VA SHIELD by an Executive Steering Committee (ESC) and Programmatic and Scientific Review Board (PSRB) (Figure 1).

The ESC provides strategic oversight for operational activities and future planning of VA SHIELD. The ESC guides the programmatic priorities and scientific agenda, assesses outcomes, and measures and reviews VA SHIELD performance and management. The overall goal of the ESC is to provide the vision for operations, advancing disease understanding and furthering the development of diagnostic, therapeutic, and preventive strategies for rapid deployment in clinical environments through research and public health surveillance.

Medical and scientific personnel wishing to access VA SHIELD data and stored biological samples apply through the VA SHIELD Coordinating Center (CC), which administratively evaluates applications for compliance before PSRB peer review. The PSRB evaluates applications for merit and biorepository impact, deciding whether and under what conditions to approve specimen and data requests submitted by investigators.

The PSRB balances the need for preservation of the biospecimen collection with the need and compelling interest to use the samples for projects that promise real impact upon the healthcare of US Veterans and society. The biospecimen collection is finite, composed at this time only of the data and biological samples available clinically; therefore, the goal is to realize the greatest scientific and public health return with the available specimens and data. The PSRB prioritizes requests to ensure that utilization of biospecimens occurs in accordance with the guidelines, protocols, and strategic objectives of VA SHIELD (Supplementary Table 1). In this regard, the quantity of the samples needed for the planned experiments influences the decision to approve a specific project.

Both the ESC and the PSRB interact with the VA SHIELD CC that directs and manages many facets of this program. Investigators who are prospective applicants for samples or data from VA SHIELD are encouraged to contact the CC. The CC is dispersed, with the Director located at the Cleveland VA Medical Center, and Associate Directors situated at the Boston, Tucson, and Boise VA Medical Centers. The VA SHIELD CC is the key operational partner, leading VA SHIELD (Figure 1) and connecting VA SHIELD to other important VA programs in the VA ORD (basic laboratory, clinical science, rehabilitation, and health services). In addition, VA SHIELD aligns VA-specific resources with other major US government institutions, including the Centers for Disease Control and Prevention (CDC), Department of Defense, National Institutes of Health, and the Food and Drug Administration. Day-to-day management of VA SHIELD is the responsibility of the VA SHIELD Director with the CC, Associate Directors, and the Co-investigators. Together, they ensure implementation of policy, develop standard operating procedures, participate in the development of the VA SHIELD strategic plan, and initiate needed communications. The VA SHIELD CC oversees the assembly of the biospecimen collection, the quality of the samples, the processing of applications, the distribution of samples, the financing of operations, and the reporting of progress. The VA SHIELD CC also convenes stakeholders to consider issues relevant to VA SHIELD, develops strategic plans to address the next emerging disease, and responds to requests to consider other duties from VA leadership. A summary of these integrated activities is discussed below.

### Operations in 2022

As of October 2022, VA SHIELD consists of 12 local specimen collection sites distributed across the US, a data processing center, and 2 federated biorepositories (Figure 1). The information flow through VA SHIELD is coordinated among each local specimen collection site, the data processing center, and the biorepository. Biologic samples are collected, processed, aliquoted, and stored at each of the local specimen collection sites. Central repositories are located at the Palo Alto and Tucson VA Medical Centers, which receive and store samples until they are distributed to approved projects. Among the 12 specimen collection sites participating in VA SHIELD, 8 sites (Atlanta, Georgia; Boise, Idaho; Cincinnati and Cleveland, Ohio; Durham, North Carolina; Palo Alto, California; St Louis, Missouri; and Tucson, Arizona) initiated specimen collection in 2020 or 2021, with 4 additional sites (Los Angeles, California; Bronx, New York; Mountain Home, Tennessee; and Houston, Texas) added in 2022. The initial sites were assembled from the VA COVID-19 response working group of committed investigators and colleagues. The sites added in 2022 were selected through competition, in response to a VA Request for Proposals.

Complete longitudinal clinical data collected as part of routine clinical care in the VA are available as of 15 October 2022 on 8 853 871 Veterans in the VA Corporate Data Warehouse (CDW). As needed for projects approved by VA SHIELD, clinical and demographic information are extracted from the CDW, including clinical course, management, therapeutics, underlying comorbidities, and vaccination history. Longitudinal clinical data, sample information, and molecular data generated from these samples were integrated and analyzed on Genomic Information System for Integrative Sciences (GenISIS), the VA research high-performance computing environment. The samples and data are interrelated following the procedure established by the Million Veteran Program (MVP) [3] with an Honest Broker intermediary to protect Veteran privacy by removing individual identifiers from the records. The process is facilitated by a nationally supported laboratory information management system (LIMS) managed by the VA SHIELD Data Processing Center in Boston, which assists investigators with data requests by assembling, cleaning, curating the data, and through the services of the Honest Broker, deidentifying the Veteran samples and the data used by investigators for their approved projects.

### Patient Consent

**Figure 2.**
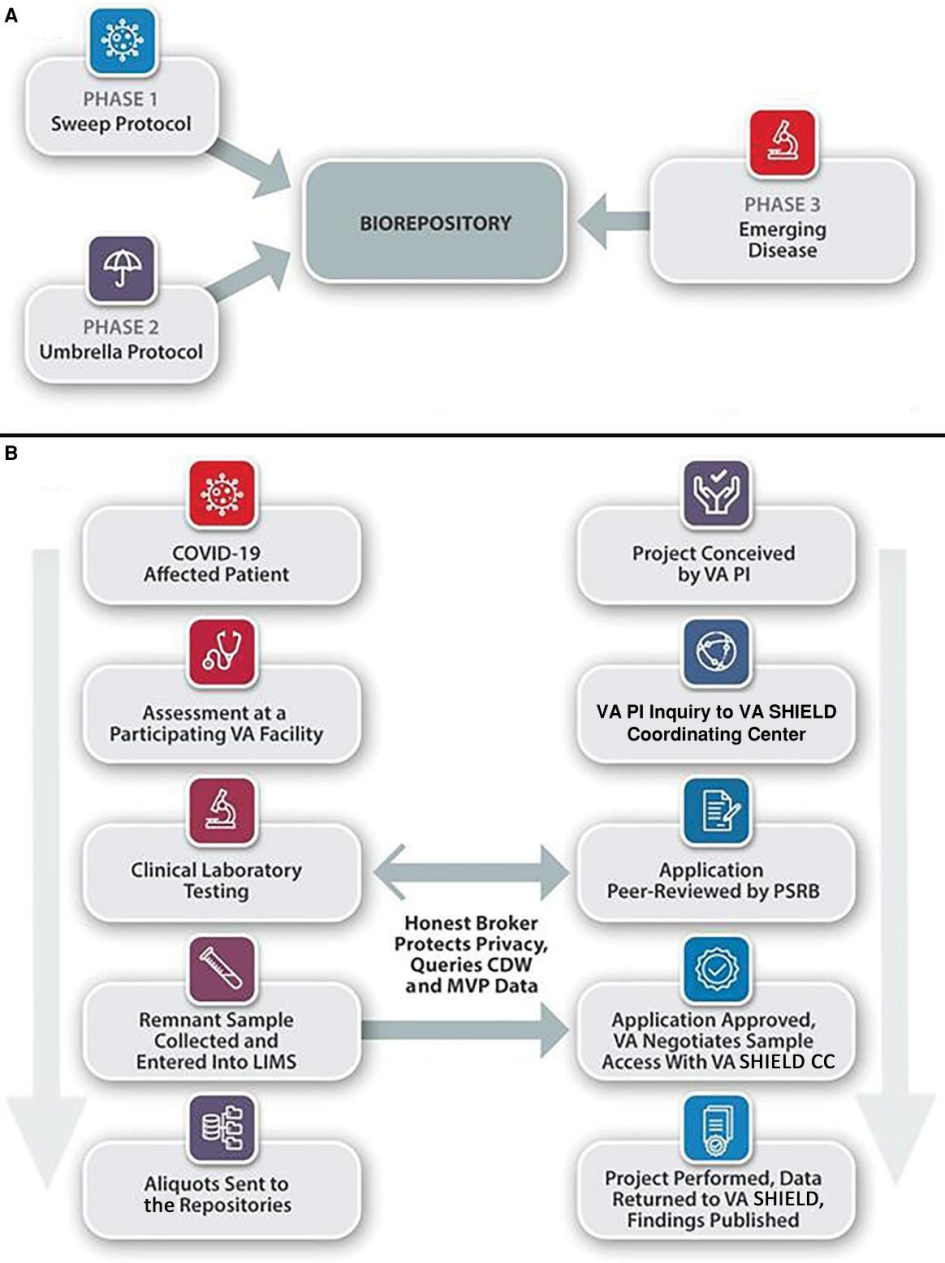
Phases for the Veterans Affairs (VA) Science and Health Initiative to Combat Infectious and Emerging Life-threatening Diseases (SHIELD) development and phase 1 biological sample and application processing. *A*, Developmental plans for VA SHIELD. The phase 1 VA Sweep protocol is underway to assemble remnant samples from severe acute respiratory syndrome coronavirus 2–affected Veterans and controls (see below). Phase 2 will be implemented in 2023 with the VA SHIELD “Umbrella” amendment to collect prospective samples from Veterans based on criteria that are being finalized. Phase 3 prepares the system for the next emerging disease that warrants a VA SHIELD response, which is underway and will become more effective upon implementation of phase 2. *B*, VA SHIELD sample flow and application approval under phase 1, the VA Sweep protocol. The steps following biological samples and data into VA SHIELD (left side) and following the development of an application for biological samples and the provision of samples to an approved project (right side) are presented. Abbreviations: CDW, Corporate Data Warehouse; COVID-19, coronavirus disease 2019; LIMS, laboratory information management system; MVP, Million Veteran Program; PI, principal investigator; PSRB, Programmatic and Scientific Review Board; SHIELD, Science and Health Initiative to Combat Infectious and Emerging Life-threatening Diseases; VA, Veterans Affairs.

Initially, each local collection site identifies residual samples that otherwise would have been discarded after clinical laboratory testing. At present, these residual samples account for most of the biological samples in VA SHIELD (Figure 2) via a novel protocol (VA Sweep), approved by the VA Central Institutional Review Board as being exempt non–human subjects research, with some samples collected as early as April 2020.

### Status of the Biospecimen Collection

As of 15 October 2022, the phase 1 VA Sweep protocol (Figure 2) has 34 157 samples from participants in VA SHIELD. Of these, 28 639 samples (83.8%) have had positive tests for SARS-CoV-2, with the remainder potentially serving as contemporaneous controls without a known SARS-CoV-2 infection. The samples include 19 788 nasopharyngeal swabs (57.9%), 9524 plasmas (27.9%), and 4284 sera (12.5%). Additional characteristics of Veteran samples and participants with demographic data available are described in Tables 2 and 3. The allowed detailed demographic and clinical information associated with the samples may be obtained as needed, through the VA Informatics and Computing Infrastructure (VINCI), for each approved project. The collection of biospecimens continues with the ongoing pandemic, providing larger numbers of residual biological samples under the VA Sweep Protocol. VA investigators may preliminarily browse the available participants, based on sample and clinical metadata, and build their cohort through the VA SHIELD GenHub sample browser (https://genhub.va.gov/sample-browser). The sample cohort of interest with accompanying data can be requested and obtained upon approval by the PSRB, after negotiation with the VA SHIELD CC (Figure 2).

**Table 2.** Characteristics of the Veterans Affairs Science and Health Initiative to Combat Infectious and Emerging Life-threatening Diseases (VA SHIELD) Samples (N = 34 157 as of 15 October 2022)

Factor	No.	(%)
COVID-19 test result		
Positive	28 639	(83.8)
Negative	3640	(10.7)
Not tested, null, or inconclusive	1878	(5.5)
Sample type		
Nasopharyngeal swab	19 788	(57.9)
Plasma	9524	(27.9)
Serum	4284	(12.5)
Other (whole blood, buffy, aspirate)	224	(0.7)

Abbreviation: COVID-19, coronavirus disease 2019.

**Table 3.** Characteristics of Veterans^a^ in the Veterans Affairs Science and Health Initiative to Combat Infectious and Emerging Life-threatening Diseases (VA SHIELD) and the VA Healthcare System

Characteristic	VA SHIELD (n = 18 375)		VA National (n = 8 853 871)	
	No.	(%)	No.	(%)
Age, y				
18–29	420	(2.3)	290 056	(3.3)
30–39	2093	(11.4)	943 001	(10.7)
40–49	2294	(12.5)	866 377	(9.8)
50–59	3480	(18.9)	1 162 250	(13.1)
60–69	4060	(22.1)	1 624 904	(18.4)
70–79	4546	(24.7)	2 365 648	(26.7)
80–89	1152	(6.3)	1 105 781	(12.5)
≥90	325	(1.8)	484 579	(5.5)
Unknown	5	(0.0)	11 275	(0.1)
Sex				
Male	15 168	(82.5)	7 777 111	(87.8)
Female	3202	(17.4)	1 074 177	(12.1)
Unknown	5	(0.0)	2583	(0.0)
Race/ethnicity				
White	9725	(52.9)	5 575 759	(67.7)
Black or African American	4605	(25.1)	1 351 620	(15.7)
Hispanic or Latino	1579	(8.6)	546 528	(1.0)
Asian	153	(0.8)	102 304	(0.9)
Native Hawaiian or Pacific Islander	134	(0.7)	71 758	(1.2)
American Indian or Alaska Native	133	(0.7)	68 224	(0.8)
Unknown	2046	(11.1)	1 137 678	(12.8)
Geographic location^b^				
Northeast	741	(4.0)	1 111 941	(12.6)
Midwest	2780	(15.1)	1 664 906	(18.8)
South	11 763	(64.0)	3 836 567	(43.3)
West	2775	(15.1)	1 851 772	(20.9)
Other	316	(1.7)	388 685	(4.4)
COVID-19 test result				
Ever tested positive	16 125	(87.8)	447 085	(5.0)
Never tested positive (recorded)	2250	(12.2)	8 406 786	(95.0)
COVID-19 vaccination status^c^				
Vaccinated and never tested positive for COVID	1434	(7.8)	3 656 140	(41.3)
Vaccinated before COVID-19 infection (breakthrough infection)	6921	(37.7)	171 249	(1.9)
Vaccinated after COVID-19 infection	3035	(16.5)	79 258	(0.9)
No record of vaccination in VA EHR	6564	(35.7)	4 808 767	(54.3)
COVID-19 severity (for COVID-19 positive)				
Mild (did not require hospitalization)	11 964	(74.2)	368 111	(82.3)
Moderate (hospitalized but did not require ICU or ventilator)	2780	(17.2)	56 103	(12.5)
Severe (hospitalized and required ICU and/or ventilator)	1405	(8.7)	23 091	(5.2)
Died within 60 d of positive COVID-19 test	797	(4.9)	14 595	(3.3)

Abbreviations: COVID-19, coronavirus disease 2019; EHR, electronic health record; ICU, intensive care unit; SHIELD, Science and Health Initiative to Combat Infectious and Emerging Life-threatening Diseases; VA, Veterans Affairs.

a Veterans for whom demographic and clinical information is available in the laboratory information management system as of 15 October 2022.

b Geographic location is defined by United States (US) census region (https://www2.census.gov/geo/pdfs/maps-data/maps/reference/us_regdiv.pdf). The Northeast region includes Connecticut, Maine, Massachusetts, New Hampshire, New Jersey, New York, Pennsylvania, Rhode Island, and Vermont. The Midwest region includes Illinois, Indiana, Iowa, Kansas, Michigan, Minnesota, Missouri, Nebraska, North Dakota, Ohio, South Dakota, and Wisconsin. The South region includes Alabama, Arkansas, Delaware, the District of Columbia, Florida, Georgia, Kentucky, Louisiana, Maryland, Mississippi, North Carolina, Oklahoma, South Carolina, Tennessee, Texas, Virginia, and West Virginia. The West region includes Alaska, Arizona, California, Colorado, Hawaii, Idaho, Montana, Nevada, New Mexico, Oregon, Utah, Washington, and Wyoming. The final category, “other,” includes US territories or cases where the participant's zip code was unknown.

c Vaccination status is retrieved from the VA EHR only and is recorded for the first injection of one of the vaccines available in the VA.

Assays for determining the presence of a SARS-CoV-2 infection have changed over the course of the pandemic. The result from the assay used clinically for this determination is provided, meaning that the tests used vary between VA Medical Centers, likely resulting in variable rates of type I and type II errors.

### Ancillary Initiatives

SUPERNOVA (Surveillance Platform and Enteric and Respiratory Infectious Organisms at Veterans Affairs Medical Centers) is a novel network of 5 VA Medical Centers initially funded by the CDC to conduct active and passive surveillance for acute gastroenteritis. In 2020, SUPERNOVA redirected its goals to conduct active surveillance for SARS-CoV-2 variants among the US Veteran population, highlighting the replacement of the SARS-CoV-2 Alpha variant (B.1.1.7) with the Delta variant (B.1.617.2) in hospitalized US Veterans [4]. In addition, early presence of the Omicron variant (B.1.1.529) in the US was detected by SUPERNOVA. Through December 2021, the SUPERNOVA sites had collected samples from 1942 Veterans, 1235 of whom are confirmed COVID-19–positive cases. In 2022, the 5 SUPERNOVA surveillance sites [5] have maintained a collection of samples and clinical information that will remain useful to investigators and public health officials.

Other VA initiatives present opportunities to address COVID-19 pandemic research questions. VA SHIELD, through the VA SHIELD CC, has taken the initiative to integrate across programs and projects. Such initiatives include (1) VA SeqCURE, which sequences SARS-CoV-2 viral variants; (2) VA CURES-1, which tested the treatment of COVID-19 using convalescent plasma from recovered patients; and (3) EPIC3, a prospective collection of samples from patients hospitalized with COVID-19, tracking visit number and day of sample collection to observe the disease over time (Supplementary Figure 1). In addition, the MVP, which was designed for large-scale genomic studies and has served as the model for VA SHIELD sample informatics, now has survey and genetic data (genome-wide genotyping and some DNA sequencing and DNA methylation) along with serum and plasma samples for >900 000 enrolled Veterans. The data collected and generated by MVP have provided numerous important insights into COVID-19 [6–10], which complements the efforts underway by VA SHIELD. Discussions have proceeded to consider ways to incorporate the SUPERNOVA COVID-19 samples and data, a VA collaboration with the CDC that has closed enrollment, into VA SHIELD.

Finally, the VA CDW data, which are accessible through the VINCI, makes up the largest electronic medical record data collection in the US and have the potential to provide access to incisive clinical and demographic data on a massive scale.

Many physicians and scientists seized the initiative to collect materials from the beginning of the pandemic; some of these collections may be incorporated into and made available to investigators through the VA SHIELD biorepository.

### Access to VA SHIELD Biospecimen and Data Biorepository: Instructions for Prospective Investigators

VA SHIELD invites public health officials and investigators, especially VA-based scientists, to consider whether use of the collection would advance their work. To obtain samples or data, VA investigators and VA public health professionals should submit a request through the VA SHIELD website (https://genhub.va.gov/sample-browser) or by contacting VAShield@va.gov. Submitted proposals are subject to peer review by the PSRB (Figure 2*B*).

Interested scientists and public health professionals not associated with the VA are strongly encouraged to collaborate with a VA-based investigator or public health colleague, who can be the applicant of record and facilitate navigating the VA compliance requirements.

Once approved by the PSRB, details are negotiated with the CC and the samples are distributed to the VA facility associated with the applicant investigator or responsible public health official. Recipients of VA SHIELD samples are required to submit any data generated from these samples back to VA SHIELD, which will later become available to other investigators, after a (to be determined) embargo period. VA SHIELD sample recipients are required to report their progress to VA SHIELD and to make their results public in the absence of any national security issue.

The VA SHIELD samples and data are offered as a service with no policy to impose authorship or collaborative requirements upon any approved project.

### Plans for the Future and Emerging Threats

We envision VA SHIELD to progress through 3 phases (Figure 2*A*). Phase 1 is underway with the samples and data now being collected under the VA Sweep Protocol (Figure 2*B*). These residual samples are collected from routine Veteran healthcare. In 2023 VA SHIELD plans to initiate phase 2 with respect to COVID-19 by prospectively enrolling COVID-19 patients and controls under the VA SHIELD “Umbrella” amendment, which would provide biospecimens for scientists and public health officials to address questions concerning vaccination, COVID-19 sequalae, and SARS-CoV-2 virus evolution. Prospective collection and enrollment will allow for standard processing and storage across VA SHIELD sites, which will improve the data generated by the assays that can be performed. The criteria for the specific samples and subjects recruited under the “Umbrella” amendment are under discussion (Supplementary Table 2).

Phase 3 of VA SHIELD. which is already underway in its nascent form, will leverage the infrastructure established under phases 1 and 2 to remain poised for rapid response to emerging healthcare crises, so that the public health and research efforts are in their strongest possible position to contribute toward reducing morbidity and mortality. When monkeypox cases reached the US in May 2022, VA SHIELD acted to preserve samples from our impacted Veteran population, demonstrating the flexibility to quickly react to emerging threats.

There are many examples of the flexibility and potential use of the VA SHIELD infrastructure. A repository of emerging highly drug-resistant bacteria as they spread among patients within the VA system is of much interest of the current investigators. The VA has played an essential role in developing numerous interventions in response to the emergence of novel strains of methicillin-resistant *Staphylococcus aureus* and toxigenic *Clostridium difficile* early in the 21st century, but the pace of such responses could have been more precise and efficient had VA SHIELD been in place at that time. Today, such an effort supported by VA SHIELD would be poised to perform detailed genetic analyses very close to real time, determining clonality and strain type, and facilitating the performance of detailed drug susceptibility studies, the testing of novel antibacterial agents, and the provision of biospecimen samples to develop rapid molecular diagnostics, vital for precision medicine in VA healthcare.

### Impact and Direction of VA SHIELD

In an unprecedented manner, the VA has responded to COVID-19 by instituting clinical programs that have rapidly vaccinated and protected many Veterans while continuing to provide state-of-the-art healthcare during the pandemic. The VA has also assembled a scientific enterprise dedicated to combating emerging infectious and other diseases and their lethal consequences. The establishment of a rapid-response biorepository and data procurement system linked to clinical samples is a bold step forward to address current and future challenges. Similarly, the integration and cooperation of multiple contributing components within the VA transcend disciplines and boundaries, promising to usher in an era of system-wide investigation. Dedicated to the VA and its public health missions, VA SHIELD will integrate with other existing government agencies to advance our mutual scientific agendas. VA SHIELD will have long-term benefits by providing the infrastructure to develop state-of-the-art biorepositories (Supplementary Figure 1) and novel technologies to address unforeseen problems, as well as to train the next generation of young investigators in these areas. The alignment of basic science, clinical, and translational research goals under one governance has a significant advantage over models of more dispersed research coordination. By supporting the national emergency preparedness efforts, the VA through VA SHIELD better positions one of the largest integrated healthcare providers in the US to leverage lessons learned and significantly impact population health.

The scope and reach of VA SHIELD create the potential for a unique impact on biomedical research. While many biobanks and healthcare systems have been established that support discovery by the broader scientific community, VA SHIELD has an efficient data management system linked to its biorepository and is one of the few biorepository systems to link emerging disease samples with longitudinal clinical data. Other similarly positioned international biorepositories (ie, the UK Biobank; see https://www.ukbiobank.ac.uk/) [11, 12]) and national health system electronic medical record data banks (eg, Denmark, Finland) also provide important opportunities for discovery.

As the prospective sample collection expands under the “Umbrella” amendment in phase 2 (Figure 2), VA SHIELD has the potential to become a large collection with its access to a reservoir of 9 million Veterans who seek services in the VA healthcare system. Our vision is that VA SHIELD will grow in sample size, collection sites, and depth of data and specimen acquisition, standing as a unique resource, and reflecting our heterogeneous Veteran demography. By harnessing data available through the LIMS, CDW, and MVP, the VA has the unprecedented opportunity to link data regarding genetics, exposure, and disease risk, which would also provide an opportunity to test novel diagnostic platforms and therapeutics for both new and existing infectious diseases, and to be prepared for the unknown challenges to come. The existing longitudinal disease risk-factor information, records of causal processes, and outcomes data present an important opportunity to optimize prevention, diagnosis, and treatment of many difficult acute and chronic diseases. VA SHIELD exemplifies the VA mission to become an ever more effective learning healthcare system.

## Supplementary Material

ofac641_Supplementary_Data

## Supplementary Data

Supplementary materials are available at *Open Forum Infectious Diseases* online. Consisting of data provided by the authors to benefit the reader, the posted materials are not copyedited and are the sole responsibility of the authors, so questions or comments should be addressed to the corresponding author.

## Notes

***Acknowledgments.*** We are grateful to the many Veterans, VA clinicians and clinical pathologists, and VA colleagues who have responded to this pandemic by providing desperately needed clinical care and encouragement in the effort to develop VA SHIELD. VA SHIELD was conceived by R. B. R., C. T. B., H. K. K., R. M. P., J. B. H., S. P., and V. C. M. Data were organized by S. P. and E. S. P. Regulatory compliance was led by V. D., J. K. B., and H. K. K. Manuscript preparation was done by J. B. H., S. P., E. S. P., L. E., A. De., C. W. W., A. Di., P. C., M. F. M., H. K. K., and R. A. B. Pilot projects were developed by J. B. H., S. B., K. M. K., S. K., C. D. S., and V. C. M. VA SHIELD applicants are K. M. K., M. C. B. A., and C. D. S. Repositories are led by J. A. W. and M. H. The VA SHIELD Coordinating Center includes S. P., D. D. B., E. S. P., I. R., C. L. S., and R. A. B. Site sample acquisitions are being collected by J. B. H., J. A. W., A. De., C. W. W., S. B., D. H. C., I. C., K. M. K., M. C. B. A., D. D. B., V. C. M., C. D. S., D. O. B., S. T. B., J. P. M., Z. Q. Y., M. C. R. B., O. Y. M. D. R., E. B. P., E. R. M., E. P., H. M. B., I. R., D. C. S., C. L. S., and R. A. B. The VA SHIELD Working Group included J. B. H., S. P., J. A. W., A. De., C. W. W., V. D., J. K. B., J. L. C., M. H., V. C. M., S. M., E. B. P., C. L. S., C. D. S., R. M. P., and H. K. K.

***Funding sources.*** The authors received no support for this research, authorship, and/or publication of this article, other than that supplied in the course of their duties for the US Department of Veterans Affairs.
