# Supplementary material for: The US Department of Veterans Affairs Science and Health Initiative to Combat Infectious and Emerging Life-Threatening Diseases (VA SHIELD): A Biorepository Addressing National Health Threats
Source: Open Forum Infect Dis. 2022 Dec 14;9(12):ofac641. doi: 10.1093/ofid/ofac641 (PMC9801224; doi:10.1093/ofid/ofac641)
Supplement: ofac641_Supplementary_Data [file ofac641_supplementary_data.zip › Supplemental Figure 1 - text.docx]

**Supplementary Figure 1. Potential VA SHIELD Institutional Collaborations**

**
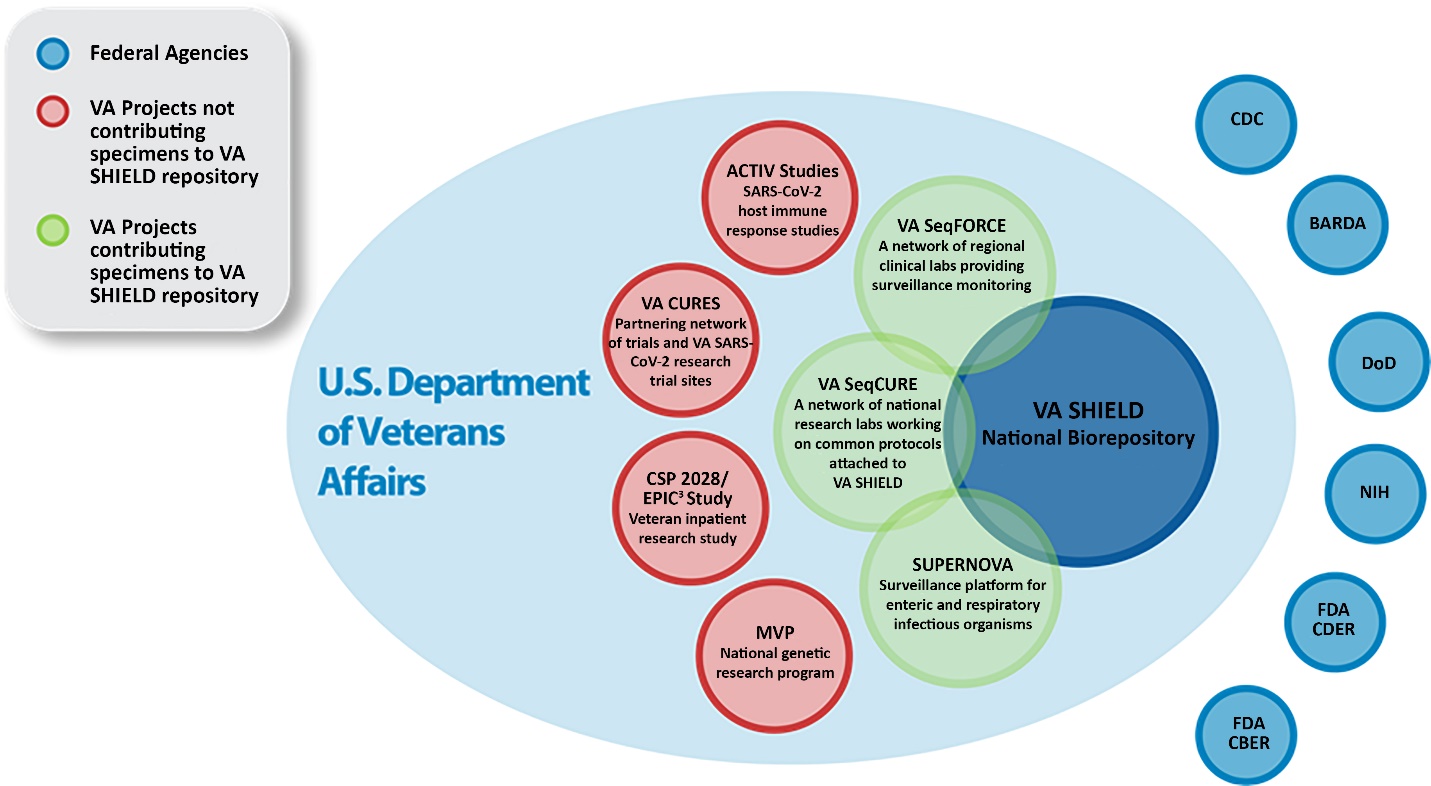
**

VA SHIELD encourages collaborative projects within the VA, such as CURES, EPIC, MVP, SeqCURE, and SUPERNOVA among others, as well as collaboration with the National Institutes of Health, the Department of Defense, and private foundations who support medical research and public health.

**ACTIV Studies**

• Accelerating COVID-19 Therapeutic Interventions and Vaccines:

Coordinated by the Foundation for the National Institutes of Health (FNIH), ACTIV studies are organized in three areas: pre-clinical, therapeutics, and prevention. The VA is contributing to preclinical activities in the Tracking Resistance and Coronavirus Evolution (TRACE) study (identifying emerging viral mutants) and in 4 ACTIV clinical trials: ACTIV-3a (TICO), ACTIV-3b (TESICO), and ACTIV-3c (VATICO), and ACTIV-4a. The primary purpose of the studies is to evaluate the safety and efficacy of multiple investigational agents aimed at modifying the host immune response to SARS-CoV-2 infection, or directly enhancing viral control to limit disease progression.

**VA CURES Program**

• VA Coronavirus Research & Efficacy Studies:

Initially established as a clinical trial master protocol framework built to support and maximize the efficiency of COVID-19 clinical trials, VA CURES serves as a partnering network of trials and VA trial sites. The program focuses on advancing clinical care of Veterans with SARS-CoV-2 infection by producing actionable data of the highest quality on strategies for novel treatments and prevention.

**CSP 2028** /**EPIC^3^ Study**

• Epidemiology, Immunology and Clinical Characteristics of COVID-19:

The study includes multiple VA facilities to reach Veterans receiving inpatient and outpatient care, and those Veterans residing in the VA’s Community Living Centers. The primary objective of the study is to describe the course of SARS-CoV-2 infection and symptomatic COVID-19 disease in Veterans who are prospectively followed for 2 years. The study aims to investigate determinants of infection, disease, and the immune response among enrolled Veterans, as well as the effectiveness of treatments and vaccines against SARS-CoV-2/COVID-19. A key contribution of this study is establishing research repositories for clinical and laboratory data, a related research specimen repository, and a participant registry to support future studies of COVID-19 and other health conditions. The study’s longer-term goal is to provide the lessons learned from this COVID-19 pandemic to better anticipate and manage pandemics in the future for the Veterans Health Administration and the general population.

**MVP Program**

• Million Veteran Program:

The Million Veteran Program is a national research program to learn how genes, lifestyle, and military exposures affect health and illness. Since launching in 2011, over 900,000 Veterans have joined MVP, making it one of the world’s largest programs on genetics and health. MVP aims to assess COVID infection and health impacts as well as behavior and well-being impacts.

**VA SeqCURE Project**

• VA Sequencing Collaborations United for Research and Epidemiology:

VA SeqCURE is a network of 5 VA sites with initial funding by the American Rescue Plan Act of 2021 (Cleveland, OH; Durham, NC; Iowa City, IA; Boise, ID; and Temple, TX). The main objective of the project is to generate sequencing data to complement the variant sequencing efforts at VA clinical labs for public health surveillance.

**VA SeqFORCE Project**

• VA Clinical Sequencing Effort for Research, Clinical and Epidemiology:

VA SeqFORCE is a network of Clinical Laboratory Improvement Amendments (CLIA)-certified VA laboratories. Founded in March 2021, VA SeqFORCE is tasked with studying and tracking variant COVID-19 strains within the VA population. Sequence data obtained by the project is used for clinical management and epidemiological studies and is reported nationally.

**CDC and VA SUPERNOVA Project**

• Surveillance Platform for Enteric and Respiratory Infectious Organisms at the VA:

SUPERNOVA is a network of 5 Veterans Affairs Medical Centers that conduct active and passive surveillance for acute gastroenteritis, with laboratory-confirmed testing of various pathogens, including norovirus and SARS-CoV-2. SUPERNOVA is effectively an endemic disease surveillance system that provides data to estimate the prevalence and incidence of norovirus in adults. Ongoing surveillance using this platform will allow for characterization of the pathogen distribution and serologic response over time.

**Federal Agencies:** CDC, Center for Disease Control; BARDA, Biomedical Advanced Research and Development Authority; DoD, Department of Defense; NIH, National Institutes of Health; FDA, Food and Drug Administration; CDER, Center for Drug Evaluation and Research; CBER, Center for biologics Evaluation and Research.
