# Supplementary material for: The US Department of Veterans Affairs Science and Health Initiative to Combat Infectious and Emerging Life-Threatening Diseases (VA SHIELD): A Biorepository Addressing National Health Threats
Source: Open Forum Infect Dis. 2022 Dec 14;9(12):ofac641. doi: 10.1093/ofid/ofac641 (PMC9801224; doi:10.1093/ofid/ofac641)
Supplement: ofac641_Supplementary_Data [file ofac641_supplementary_data.zip › Supplemental Table 1.docx]

**Supplementary Table 1**.

**Criteria for Assessing Investigator-Initiated Proposals to Use VA SHIELD Samples and Data**

| - Scientific excellence - Feasibility - Capabilities of the research team and their work environment - Impact of the proposed study upon the collection of biospecimens in VA SHIELD - Relevance to the VA healthcare mission and to human health writ large - Impact and timeliness of the study upon the understanding of or response to an emerging disorder - Compliance with US law and VA rules and regulations. |
| --- |
