# Supplementary material for: The US Department of Veterans Affairs Science and Health Initiative to Combat Infectious and Emerging Life-Threatening Diseases (VA SHIELD): A Biorepository Addressing National Health Threats
Source: Open Forum Infect Dis. 2022 Dec 14;9(12):ofac641. doi: 10.1093/ofid/ofac641 (PMC9801224; doi:10.1093/ofid/ofac641)
Supplement: ofac641_Supplementary_Data [file ofac641_supplementary_data.zip › Supplemental Table 2.docx]

**Supplementary Table 2.**

**Possible Umbrella Amendment Criteria for Selection of Samples and Subjects**

| - Any US Veteran testing positive for a concurrent SARS-CoV-2 infection and invited to provide nasopharyngeal swabs in carrier media, peripheral blood, and questionnaire responses - Any US Veteran population survey participants enrolled at 1 of the 12 collection sites for blood samples and questionnaire - Any US Veterans ascertained by a VA clinician to have sequalae of SARS-CoV-2 infection - Any US Veterans ascertained by a VA clinician to have a complication from a vaccination, especially those designed to provide immunity against SARS-CoV-2 - Any sample collections being assembled under the Umbrella protocol that are extended to include Veteran family members and VA personnel - Any emerging biomedical threat warranting the focused resources of VA SHIELD for the specific purpose of collecting biological samples and data (a Phase 3 activity), as determined by the VA Office of Research and Development (ORD), which could possibly include major morbidity and mortality from:   - New and emerging biothreats including radiation and chemical exposures   - A new, dangerous viral pandemic   - Deadly antimicrobial antibiotic resistance   - A toxin in foods, health food supplements, or other mass-marketed consumer products |
| --- |
