# Supplementary figures and images for: The US Department of Veterans Affairs Science and Health Initiative to Combat Infectious and Emerging Life-Threatening Diseases (VA SHIELD): A Biorepository Addressing National Health Threats
Source: Open Forum Infect Dis. 2022 Dec 14;9(12):ofac641. doi: 10.1093/ofid/ofac641 (PMC9801224; doi:10.1093/ofid/ofac641)

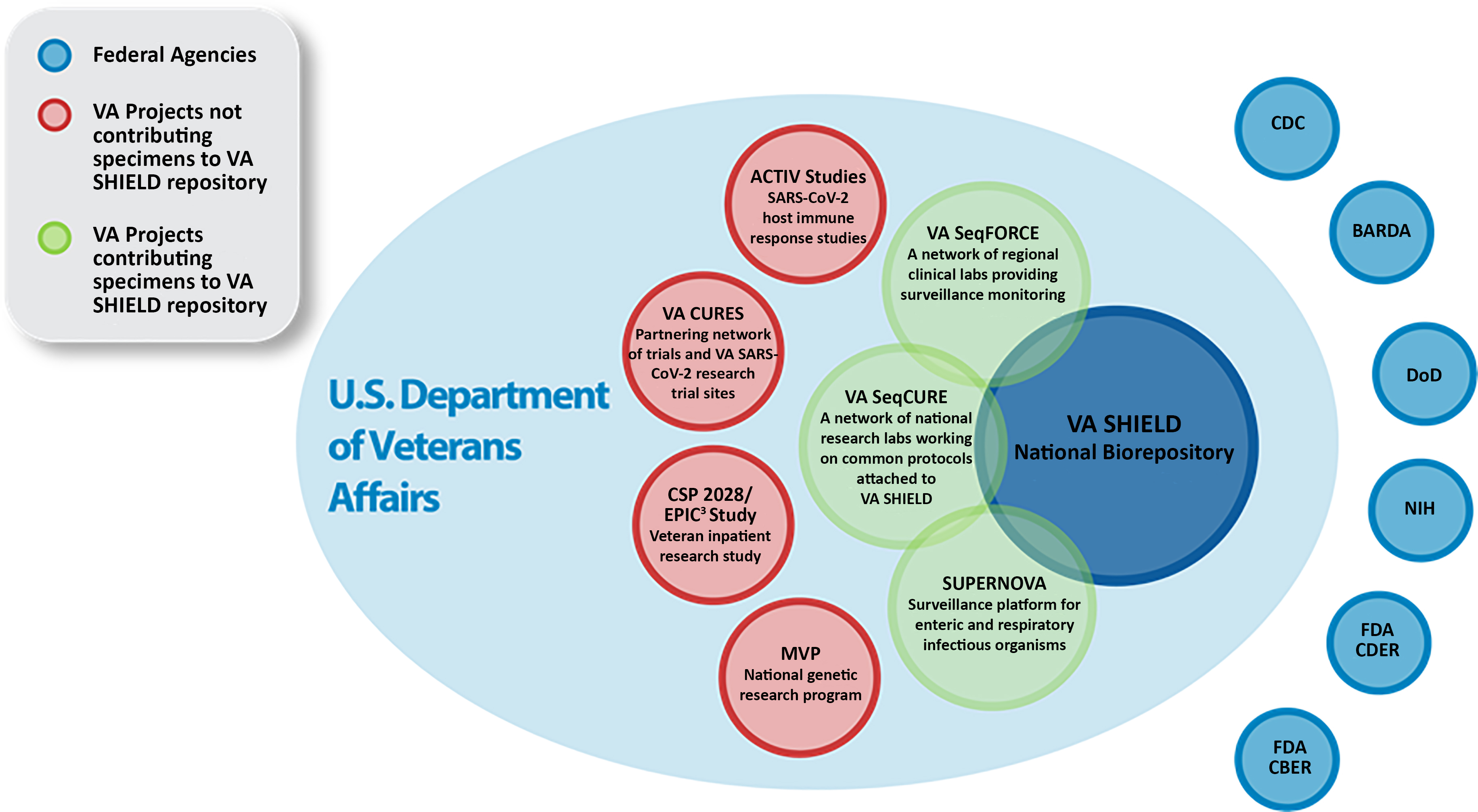

Supplement: ofac641_Supplementary_Data [file ofac641_supplementary_data.zip › Supplemental Figure 1.jpg]
